# Latest Advances in Active Materials for Food Packaging and Their Application

**DOI:** 10.3390/foods12224055

**Published:** 2023-11-07

**Authors:** Yongli Jiang, Yifeng Zhang, Yun Deng

**Affiliations:** 1 Faculty of Food Science and Engineering, Kunming University of Science and Technology, Kunming 650500, China; yongli_jiang0617@163.com; 2 Department of Food Safety and Health, School of Advanced Agriculture Sciences, Peking University, Beijing 100871, China; zhangyifeng@pku.edu.cn; 3 School of Agriculture and Biology, Shanghai Jiao Tong University, Shanghai 200240, China

## 1. Introduction

Food packaging plays a pivotal role in preserving the quality and safety of food products while extending their shelf life. Over time, the field of food packaging has evolved from simple containment to sophisticated systems that actively interact with the packaged food, which has led to the emergence of active materials. The practical applications of active materials in food packaging are nothing short of transformative. From fresh produce to meat, poultry, bakery, snacks, and beverages, active materials have become integral to various parts of the food industry. This innovation has transformed the way in which we view food packaging, and allows it to be not only a vessel but a dynamic component in the quest to keep our food fresh and safe.

In addition to their primary functions (e.g., extending the shelf life of food and increasing its value), active packaging systems perform some additional functions in preserving freshness, quality, and safety. For example, certain active packaging materials are designed to slow down the rate of respiration, inhibit microbial growth, or mitigate moisture migration. The functionality of active packaging materials hinges on their active substances, which may offer properties such as releasing, absorbing, blocking, or buffering effects. Broadly speaking, active packaging can be categorized into two main types: non-migratory active packaging and active releasing packaging. Non-migratory active packaging typically involves scavengers engineered to eliminate unwanted components from the packaging environment without intentional migration. The common functions of non-migratory active packaging systems within food products include their abilities to act as oxygen scavengers, moisture scavengers, and ethylene absorbers. In contrast, active releasing packaging primarily encompasses emitters that facilitate the controlled migration of desired substances into the packaging environment, positively impacting the food product. Active releasing packaging systems encompass carbon dioxide emitters, antimicrobial packaging, and antioxidant packaging [1]. 

The ongoing development of new active packaging systems is being propelled by technological advancements, resulting in higher standards of food safety and quality, as well as reduced waste and increased sustainability. Researchers are exploring novel antimicrobial agents, antioxidants, and biobased materials. Nanotechnology has allowed the creation of nanocomposites with remarkable mechanical strength, gas barrier properties, water repellency, and antimicrobial and scavenging activities. Nanotechnology applications are expanding to create multifunctional nanocomposites that are capable of addressing multiple packaging challenges simultaneously. These innovations promise to revolutionize packaging, leading to materials with enhanced barrier properties and a reduced environmental impact. Moreover, these materials can be integrated into chemical and biological sensors, enabling the development of rapid and sensitive devices for assessing freshness and detecting allergens, toxins, or pathogenic contaminants. These innovations are not confined to the laboratory and are being translated into tangible benefits for consumers and the food industry as a whole. Through the leveraging of these breakthroughs, the performance of food packaging is being enhanced in ways that were previously unimaginable.

## 2. Active Materials for Food Packaging

Active materials represent a revolutionary leap in food packaging technology, offering enhanced preservation and safety benefits. The trend toward sustainability and eco-friendly packaging has led to innovations in biobased packaging materials. Researchers are actively exploring novel sourcing and processing techniques and biopolymer blends to optimize both the performance and the sustainability of packaging. Biobased materials show great potential for replacing conventional petroleum-based materials in packaging applications due to their abundance, biocompatibility, biodegradability, and non-toxic nature. These biopolymer-based packaging materials include polysaccharides (e.g., chitosan, cellulose and its derivatives, starch, etc.), lipids and proteins, and some other specific biopolymers (e.g., polylactic acid or polyvinyl alcohol) [2]. The potential of different natural and synthetic polymers for their film- and coating-forming abilities has been widely studied, considering their abundance, biological properties (being biocompatible, biodegradable, and non-toxic), and morphological and physiological features. 

In addition to the intrinsic features of biomaterials, the introduction of different functional groups, the addition of exogenous materials, and the grafting of bioactive polymers offer additional functionalities and bioactivity to these biopolymers. Antimicrobial and antioxidant agents, for instance, are incorporated into packaging materials to inhibit microorganism growth and prevent food oxidation. In the realm of antimicrobial and antioxidant agents, efforts are currently being focused on the development of novel main polymers with broad-spectrum activities or the harnessing of various bioactive components sourced either from natural agents (bacteriocins, essential oils, polyphenols, etc.) or from synthetic agents, whether organic or inorganic (Ag, ZnO, TiO_2_ nanoparticles, synthetic antibiotics, etc.) [3]. Another area of intense research interest concerns the methods employed in determining antimicrobial and antioxidant activities. These methods vary, from including sachets with volatile agents inside existing packaging to incorporating the active agent directly into biopolymers, and from coating or grafting antimicrobial and antioxidant substances onto polymeric surfaces to the use of intrinsic polymers or pads. Recently, essential oils were incorporated into edible films to introduce active edible films with antimicrobial and antioxidant properties.

Despite active materials holding significant promise for food packaging applications, there are several critical factors to address in order to attract investors and meet market demand. For instance, safety assessments, as well as the in vivo degradation and safety of the metabolites produced, should be further investigated. In addition, the high cost associated with certain biobased materials necessitates process optimization and the exploration of alternative raw materials for their production. Nevertheless, pressure from regulatory bodies and consumers alike will shape the future of active packaging materials toward novel, cost-efficient, biodegradable materials that can ensure food safety and quality and a longer shelf life.

## 3. Active Packaging Applications 

The practical applications of active materials in food packaging are extensive and continuously expanding. Oxygen scavengers, antimicrobial films, and smart labels that monitor temperature and freshness have been widely adopted. In fresh produce packaging, active materials, such as ethylene scavengers, moisture-control films, and antimicrobial coatings, are commonly employed to maintain product freshness. Meat and poultry packaging developments benefit from active solutions that prevent spoilage and reduce microbial contamination. Active packaging solutions for bakery and snack products include oxygen-absorbing packaging, which helps to preserve product crispness, and anti-fungal coatings to prevent mold growth. Beverages benefit from active packaging through oxygen-barrier materials that protect the flavor and quality of the liquid contents. Additionally, UV-blocking coatings prevent light-induced degradation [4]. 

The commercial adoption of active packaging is on the rise, driven by consumer demand for a longer shelf life and safer products. Major food manufacturers and retailers are increasingly integrating active packaging solutions into their product lines, marking a significant shift in the industry landscape.

## 4. Active Packaging and Safety 

Ensuring food safety is of paramount concern in the food packaging industry, and active packaging plays a critical role in enhancing the safety of packaged products. Active packaging materials are strategically designed to actively interact with the packaged food, mitigating the risks associated with microbial contamination, oxidation, and other factors that could compromise food safety. This interaction does not simply include passive containment; rather, it is a dynamic process that actively contributes to food preservation and safety.

The safety of packaging materials themselves is equally important. Active packaging materials must undergo a rigorous evaluation to ensure that they do not compromise food safety or public health. The safety of these materials is a top priority for both regulatory agencies and manufacturers. Regulatory agencies worldwide have established stringent guidelines and standards to assess the safety of active packaging materials. These regulations ensure that materials used in contact with food do not pose health risks to consumers. Next, extensive migration testing is conducted, to determine whether any components from the active packaging materials leach into the food. Active packaging materials are also scrutinized for their environmental impact. Biodegradability and sustainability are key considerations, aligning with the global efforts to reduce plastic waste and minimize the environmental footprint of packaging materials [5]. The synergistic combination of enhanced food safety and packaging material safety underscores the pivotal role of active packaging in modern food-packaging practices.

## 5. Future Trends

Looking ahead, the future of active materials in food packaging holds exciting prospects and unique challenges. Sustainability is a central theme, with biodegradable and compostable materials gaining prominence. The invention of new production processes, as well as the introduction of new active materials with a higher stability and improved physico-chemical properties, will represent the subject of further research. Advances in active packaging with a low carbon footprint are on the horizon, driven by innovations in production methods and materials. Intelligent packaging is another frontier, offering real-time monitoring capabilities to consumers. Sensors and data-sharing technologies provide information on product freshness, safety, and authenticity, revolutionizing the consumer experience [6]. Additionally, addressing challenges such as regulatory compliance is essential to the continued success of active materials in food packaging.

In conclusion, active materials for food packaging represent a dynamic and evolving field that addresses the critical requirements of food preservation, safety, and sustainability. While some challenges remain, they serve as catalysts for innovation, propelling the industry toward safer, more efficient, and environmentally conscious packaging methods. This Special Issue aims to showcase innovative research that combines material sciences, chemistry, and engineering to create packaging solutions that extend shelf life, minimize spoilage, and guarantee food quality. By fostering collaboration between academia and industry, we aspire to catalyze advancements that bolster food quality, prolong shelf life, and address crucial sustainability challenges.
